# Low Frequency Electromagnetic Field Conditioning Protects against I/R Injury and Contractile Dysfunction in the Isolated Rat Heart

**DOI:** 10.1155/2015/396593

**Published:** 2015-04-15

**Authors:** Dariusz Bialy, Magdalena Wawrzynska, Iwona Bil-Lula, Anna Krzywonos-Zawadzka, Mieczyslaw Wozniak, Virgilio J. J. Cadete, Grzegorz Sawicki

**Affiliations:** ^1^Department and Clinic of Cardiology, Medical University of Wroclaw, 50-556 Wroclaw, Poland; ^2^Department of Medical Emergency, Medical University of Wroclaw, 51-616 Wroclaw, Poland; ^3^Department of Clinical Chemistry, Medical University of Wroclaw, 50-556 Wroclaw, Poland; ^4^Department of Pharmacology, College of Medicine, University of Saskatchewan, 107 Wiggins Road, Saskatoon, SK, Canada S7N 5E5; ^5^Faculty of Pharmacy, University of Montreal, Montreal, QC, Canada H3T 1J4

## Abstract

Low frequency electromagnetic field (LF-EMF) decreases the formation of reactive oxygen species, which are key mediators of ischemia/reperfusion (I/R) injury. Therefore, we hypothesized that the LF-EMF protects contractility of hearts subjected to I/R injury. Isolated rat hearts were subjected to 20 min of global no-flow ischemia, followed by 30 min reperfusion, in the presence or absence of LF-EMF. Coronary flow, heart rate, left ventricular developed pressure (LVDP), and rate pressure product (RPP) were determined for evaluation of heart mechanical function. The activity of cardiac matrix metalloproteinase-2 (MMP-2) and the contents of coronary effluent troponin I (TnI) and interleukin-6 (IL-6) were measured as markers of heart injury. LF-EMF prevented decreased RPP in I/R hearts, while having no effect on coronary flow. In addition, hearts subjected to I/R exhibited significantly increased LVDP when subjected to LF-EMF. Although TnI and IL-6 levels were increased in I/R hearts, their levels returned to baseline aerobic levels in I/R hearts subjected to LF-EMF. The reduced activity of MMP-2 in I/R hearts was reversed in hearts subjected to LF-EMF. The data presented here indicate that acute exposure to LF-EMF protects mechanical function of I/R hearts and reduces I/R injury.

## 1. Introduction

Low frequency electromagnetic fields (LF-EMFs) have been used for over 30 years in orthopaedics to enhance bone healing [1–4] and also in skin lesion repair [5] and neoangiogenesis [6]. Several biological systems have been studied, with particular focus on the cardiovascular and central nervous systems as primary targets of LF-EMF. This is primarily due to shared common characteristics between these two systems, such as high electrical activity and sensitivity to induced electrical currents, which make these systems potential targets of electromagnetic fields. Although several human studies have evaluated the effects of long- and short-term exposure to LF-EMF on the cardiovascular system, the contradictory results reported (ranging from increased cardiovascular risk to increased cardioprotection and the absence of any effect) further add to the controversial discussion on the effects of LF-EMF on biological systems.

Despite the numerous studies, the underlying mechanisms regarding the interaction between electromagnetic fields and biological systems remain unknown. Little is also known about the acute effects of LF-EMF. The study of the effects of LF-EMF on the cardiovascular system demonstrated protection against myocardial infarction (MI) [7]. Similarly, data from Ma and colleagues [8] suggest that LF-EMFs inhibit the generation of reactive oxygen species, such as nitric oxide and peroxynitrite, thereby protecting cardiomyocytes from I/R and oxidative damage. The protective effects of LF-EMF are further supported by a study examining the effects of LF-EMF on skin wound healing which suggests that LF-EMF stimulates endogenous antioxidant systems. A recent study by Kim and colleagues [9], involving both teenaged and adult subjects, suggests that acute exposure to LF-EMF has no physiological effects. Taken together, these observations strongly suggest a potential protective role of LF-EMF against cardiac injury, with minimal physiological alterations.

The purpose of this study was to evaluate the protective effects of LF-EMF in a model of* ex vivo* cardiac I/R. We demonstrate that LF-EMF alone, when applied prior to, during, and after the ischemic insult, protects the heart against I/R-induced cardiac contractile dysfunction and heart injury.

## 2. Materials and Methods

All procedures were performed in conformity with “Guidelines and Authorization for the Use of Laboratory Animals” (Polish Government, Ministry of Health).

### 2.1. Retrograde Isolated Rat Heart Perfusions

Male Wistar rats (300 g to 350 g) were anaesthetized with sodium pentobarbital (60 mg/kg i.p.). Once surgical plane was achieved, hearts were rapidly excised and rinsed by immersion in ice-cold Krebs-Henseleit buffer, followed by cannulation of the aorta and beginning of the retrograde perfusion at constant flow (10 mL/min). Spontaneously beating hearts were perfused in the Langendorff mode at a constant pressure of 60 mmHg in a water-jacketed chamber at 37°C (EMKA Technologies, Paris, France). Krebs-Henseleit buffer at 37°C containing (in mM) NaCl (118), KCl (4.7), KH_2_PO_4_ (1.2), MgSO_4_ (1.2), CaCl_2_ (3.0), NaHCO_3_ (25), glucose (11), and EDTA (0.5) and gassed continuously with 95% O_2_/5% CO_2_ (pH 7.4) was used throughout the perfusion period.

Left ventricular pressures and heart rate were measured with the help of a water-filled latex balloon connected to a pressure transducer and inserted through an incision in the left atrium into the left ventricle through the mitral valve. The volume was adjusted at the beginning of the perfusion period to achieve an end-diastolic pressure of 10 mmHg. Coronary flow, heart rate, and left ventricular pressure were monitored using an EMKA recording system with IOX2 software (EMKA Technologies, Paris, France). Left ventricular developed pressure (LVDP) was calculated as the difference between peak systolic and diastolic pressures. The rate pressure product (RPP) was calculated as the product of heart rate and LVDP.

### 2.2. Ischemia/Reperfusion Protocol and Low Frequency Electromagnetic Field Exposure

Control hearts (aerobic control, *n* = 9) were perfused aerobically for 75 minutes. Ischemic hearts (I/R, *n* = 9), after 25 min of aerobic perfusion, were subjected to 20 minutes global no-flow ischemia (by closing of the aortic inflow line), followed by 30 minutes of aerobic reperfusion.

**Figure 1:**
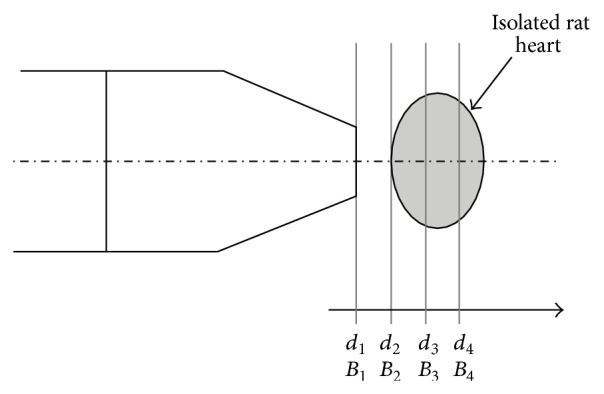
Diagram of dependence of the electromagnetic field to distance (*d*_1–4_) from the head of applicator to the isolated heart. Different electromagnetic fields are represented by *B*_1–4_. The arrow indicates increased distance and decreased electromagnetic field from the head. The estimated values of electromagnetic induction (*B*) in relation to distance (*d*) from the head of the applicator are showed in Table 1.

Low frequency electromagnetic fields (LF-EMF) were applied to a subset of hearts perfused either aerobically (*n* = 9) or subjected to I/R (*n* = 9). LF-EMF was generated by a point applicator *Z* connected to a Viofor JPS classic control unit (Viofor JPS, Poland). Magnetic field induction (*B*) varied, depending on the distance (*d*), from the inducing point applicator *Z* and averaged 500 *μ*T.  Figure 1 schematizes the dependence of the field on distance.  Table 1 summarizes the field variations (*B*_1–4_) with respect to distance (*d*_1–4_).

**Figure 2:**
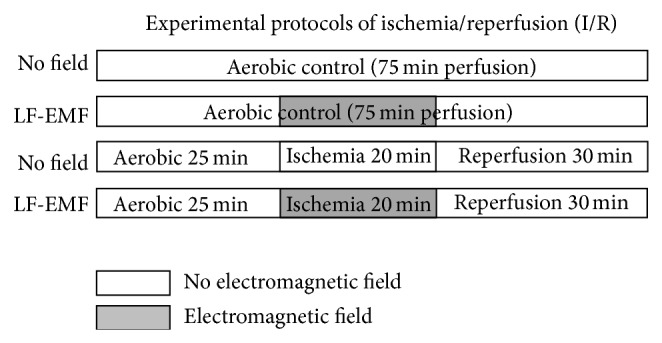
Schematic representation of the perfusion protocol for isolated rat hearts subjected to global no-flow ischemia and protected with low frequency electromagnetic field (LF-EMF).

The scheme of the experimental protocol is shown in Figure 2. After 20 min of aerobic perfusions (control hearts) or at the first minutes of reperfusion (from I/R hearts) samples of perfusates were collected for measurement of interleukin-6 (IL-6) and tropoponin I (TnI). At the end of perfusion the hearts were freeze-clamped in liquid nitrogen and used for measurement of activity of matrix metalloproteinase-2 (MMP-2).

### 2.3. Measurement Interleukin-6 and Troponin I Levels

IL-6 from coronary perfusate was measured by ELISA method using Quantikine Rat IL-6 Immunoassay (R&D Systems, USA). TnI from coronary effluent was measured by ELISA method using Rat TnI, fast cardiac muscle ELISA kit from Wuhan EIAaB Science Co. (Wuhan, China). Before biochemical analysis all perfusates were concentrated in Amicon Ultra concentrating vessels (EMD Millipore, Billerica, MA, USA). The final volume of concentrate was measured by gravimetry and adjusted to the same final volume for each sample (500 *μ*L).

### 2.4. Measurement of MMP-2 Activity

Gelatin zymography was performed as previously described [4, 10, 11]. Briefly, homogenates from heart preparations containing 10 *μ*g of protein were applied to 8% polyacrylamide gel copolymerized with 2 mg/mL gelatin. After electrophoresis, gels were rinsed three times for 20 minutes in 2.5% Triton X-100 to remove SDS. The gels were then washed twice in incubation buffer (50 mM Tris-HCl, 5 mM CaCl_2_, 150 mM NaCl, and 0.05% NaN_3_) for 20 minutes at room temperature and incubated in incubation buffer at 37°C for 24 hours. The gels were stained using 0.05% Coomassie Brilliant Blue G-250 in a mixture of methanol : acetic acid : water (2.5 : 1 : 6.5, v : v : v) and destained in aqueous solution of 4% methanol : 8% acetic acid (v : v). Developed gels were scanned with GS-800 calibrated densitometer and MMP-2 activity was measured using Quantity One 4.6 software (Bio-Rad, Hercules, CA, USA).

### 2.5. Statistical Analysis

Data from contractility measurements and biochemical studies were analyzed with ANOVA and Kruskal-Wallis post hoc analysis or Student's *t*-tests. A *P* < 0.05 indicated statistical significance. Data are presented as the mean ± SEM.

## 3. Results

### 3.1. Effect of Low Frequency Electromagnetic Field on Cardiac Hemodynamic Parameters

**Figure 3:**
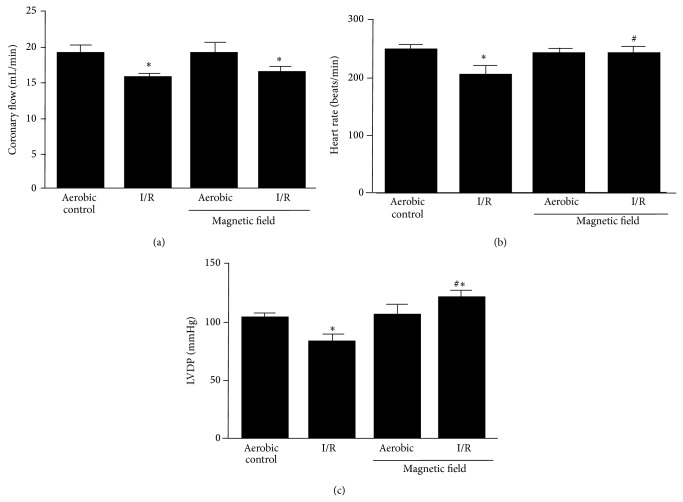
Effect of low frequency electromagnetic field (LF-EMF) on hemodynamic parameters on hearts subjected to global no-flow ischemia and reperfusion. Effect of LF-EMF on (a) coronary flow, (b) heart rate, and (c) LVDP. *n* = 9 heart preparations per group; ^∗^ *P* < 0.05 versus aerobic control; ^#^ *P* < 0.05 versus I/R alone.

In order to evaluate the effect of LF-EMF on isolated perfused rat hearts subjected to no-flow ischemia followed by reperfusion, hemodynamic parameters measured during and at the end of the perfusion protocol were analyzed (Figure 3). As previously reported [12], subjecting hearts to an ischemia/reperfusion (I/R) protocol results in a significant decrease in coronary flow (Figure 3(a)) concomitant with decreases in heart rate (Figure 3(b)) and left ventricular developed pressure (LVDP) (Figure 3(c)), in comparison to aerobically perfused hearts. Subjecting aerobically perfused hearts to an induced LF-EMF did not affect any of the measured parameters (Figures 3(a)–3(c)). However, when hearts were subjected to I/R, while having no effect on coronary flow (Figure 3(a)), LF-EMF prevented the I/R-induced decrease in heart rate and LVDP (Figures 3(b) and 3(c), resp.).

### 3.2. Effect of LF-EMF on Cardiac Contractility and Markers of Cardiac Tissue Injury and Outcome

**Figure 4:**
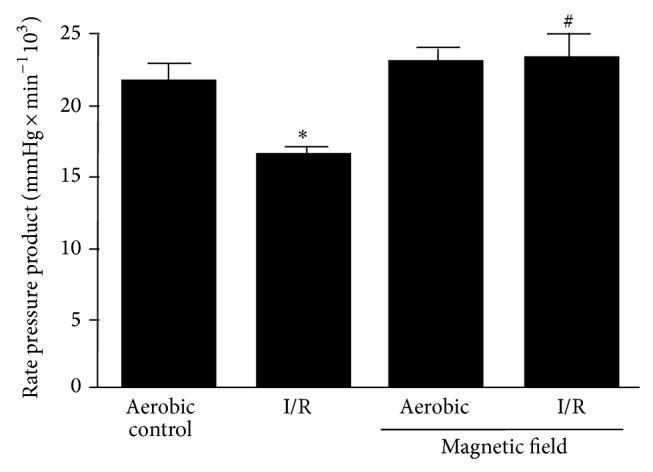
Effect of low frequency electromagnetic field (LF-EMF) on heart contractility subjected to ischemia/reperfusion (I/R) injury. *n* = 9 heart preparations per group; ^∗^ *P* < 0.05 versus aerobic control; ^#^ *P* < 0.05 versus I/R alone.

Cardiac contractility was evaluated by rate pressure product (RPP) at the end of the perfusion protocol (Figure 4). As expected (taking into account that RPP is the product of heart rate and LVDP), LF-EMF did not alter RPP in aerobically perfused hearts but prevented the reduction of RPP in I/R hearts (Figure 4).

**Figure 5:**
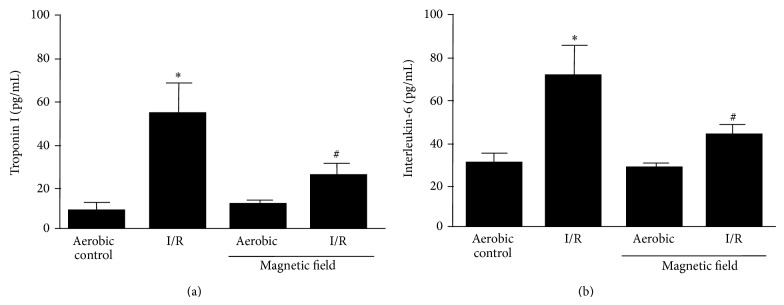
The effect of low frequency electromagnetic field (LF-EMF) on release of troponin I (TnI) (a) and interleukin-6 (IL-6) from hearts subjected to ischemia/reperfusion (I/R) injury. *n* = 9 heart preparations per group; ^∗^ *P* < 0.05 versus aerobic control; ^#^ *P* < 0.05 versus I/R alone.

The levels of TnI (marker of heart cardiac damage in acute coronary syndromes [13]) and IL-6 (marker of inflammation [14]) were measured in perfusates to determine cardiac tissue injury. Similar to what is observed clinically, the levels of both TnI and IL-6 were significantly increased in the perfusates of hearts subjected to I/R (Figure 5). When hearts were subjected to I/R in the presence of LF-EMF the levels of TnI and IL-6 in the perfusates were significantly decreased in comparison to I/R hearts but not significantly different from LF-EMF aerobic hearts (Figure 5).

### 3.3. Effect of LF-EMF on MMP-2 Activity in I/R Hearts

MMP-2 significantly contributes to heart I/R injury by degrading cardiac contractile proteins [15–18]. Further, MMP-2 contributes to the damage of endothelium in I/R hearts and consequent increase in protein release [19, 20], including MMP-2 itself.

**Figure 6:**
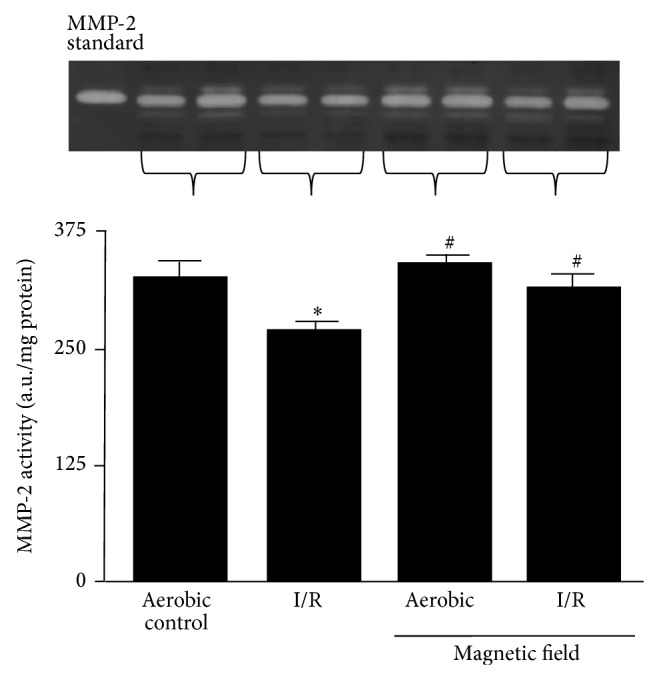
The effect of low frequency electromagnetic field (LF-EMF) on matrix metalloproteinase-2 (MMP-2) activity in hearts subjected to ischemia/reperfusion (I/R) injury. *n* = 9 heart preparations per group; ^∗^ *P* < 0.05 versus aerobic control; ^#^ *P* < 0.05 versus I/R alone.

MMP-2 activity was significantly decreased in I/R hearts, in comparison to aerobic controls (Figure 6), likely due to endothelial damage and increased protein release. LF-EMF did not affect MMP-2 activity in aerobically perfused hearts and reduced the decrease in MMP-2 tissue activity induced by I/R (Figure 6).

## 4. Discussion

Despite significant technological and pharmacological advances in the management of heart disease, novel therapeutic alternatives are desired to prevent cardiac tissue damage. Although still controversial, the use of low frequency electromagnetic field (LF-EMF) as a nonpharmacological, noninvasive protective intervention against ischemia/reperfusion- (I/R-) induced cardiac injury is a promising technique that deserves further attention to establish the mechanisms underlying potential cardioprotective effects. This study demonstrates that LF-EMFs protect against cellular damage and preserve mechanical function in hearts subjected to I/R. In addition, this study provides additional support to earlier studies demonstrating LF-EMFs could be of particular clinical relevance in situations in which onset of ischemia and/or reperfusion are controlled, such as CABG surgery and reperfusion therapy.

A number of health concerns have been raised relating to chronic environmental LF-EMF exposure. Several studies, including human studies, have reported contradictory observations regarding the relationship between LF-EMF and oxidative stress [21]. Goraca and colleagues [22] reported a decrease in antioxidant capacity in hearts of rats subjected to chronic LF-EMF exposure. Similarly, prolonged exposure to LF-EMF enhanced free radical generation in the brain and retina of rats leading to the reduced antioxidant defense capacity and increased lipid peroxidation [23–25]. Recently, it has been shown that the exposure of blood samples to LF-EMF induced changes in ROS production in both stimulated and nonstimulated neutrophils [25]. Taken together, these observations support the growing concerns regarding chronic exposure to LF-EMFs. Indeed, it appears that chronic exposure to LF-EMF can have a negative impact on the antioxidant capacity.

Despite the potential deleterious effects of chronic exposure to LF-EMF, the evidence suggests short-term exposure to LF-EMF has little or no effect on healthy subjects [9]. Raggi and colleagues demonstrated a significant reduction of blood malondialdehyde levels (free radicals marker) following LF-EMF exposure [21]. LM-EMFs were also found to induce an increase of glutathione peroxidase activity and a decrease in malondialdehyde concentration in liver and serum [26]. Such evidence suggests an effect of LM-EMF on the development of protective antioxidant activity.

Since short-term exposure to LF-EMF has no effect on healthy subjects, or if any, potentially protective, the use of acute LF-EMF as a therapeutic tool to protect against injury is currently being explored. Ma and colleagues demonstrated acute exposure to LF-EMF protects isolated cardiomyocytes from I/R-induced cell death by mediating ROS production and maintaining the NO/ONOO^−^ balance [8]. Although Ma and colleagues did not examine cardiomyocyte contractility, their data suggests a possible mechanism behind the observed LF-EMF cardioprotection. ROS generation can lead to protein and lipid oxidation. At the level of contractile proteins, we have previously demonstrated that cardiac contractile protein nitration and nitrosylation in response to I/R increase its degradation [27]. Consequently, a reduction in ROS production (as described by Ma and colleagues) would result in decreased contractile protein degradation and preservation of cardiac function as previously shown [27–31]. In addition, since MMP-2 can be activated by ROS, a reduction in ROS production would attenuate MMP-2 activation, contribute to the preservation of endothelial integrity [30], and further reduce cardiac contractile protein degradation and consequent cardiac contractile dysfunction induced by I/R injury. These effects would explain our observations relating to MMP-2 activity in response to I/R. Under conditions of acute stress, such as I/R, endothelial integrity can be compromised leading to increased protein release [19, 20]. In our model we observed decreased MMP-2 tissue activity in the I/R group indicating that endothelial integrity was compromised and MMP-2 was released. When I/R hearts were exposed to LF-EMF, MMP-2 activity was similar to that of controls suggesting that a consequence of LF-EMF cardioprotection is the preservation of endothelial integrity.

The regulation of calcium homeostasis is another potential mechanism modulating the observed cardioprotective effects of LF-EMF. Increased Ca^2+^ levels have been observed in cardiac ventricular cells in response to LF-EMF exposure [32]. LF-EMF can induce depolarization of the cell membrane followed by an increase of Ca^2+^ and expression of neurofilament protein [33]. In addition, LF-EMF stimulates the differentiation of embryonic stem cells into cardiomyocytes. The modulation of proliferation and cardiac differentiation observed in LF-EMF/Ca^2+^ exposed cells correlates with induced changes in intracellular Ca^2+^ accumulation and stimulation of signaling cascade pathways [10]. In our model of I/R, calcium deregulation is a relevant mechanism contributing to the development of I/R injury. LF-EMF can have distinct effects on calcium homeostasis, either protective or deleterious, which appear to be cell specific (for review see [11]). Although the dissection of the effects of LF-EMF on calcium homeostasis was not the focus of this study, it is possible that part of the cardioprotective effect that we observed was due to LF-EMF-induced alterations on calcium homeostasis and signaling.

In addition, we observed a significant protection of cardiac function in hearts exposed to short exposure of LF-EMF. It is known that isolated rat hearts subjected to ischemia exhibit decreased maximal force generation, as well as increased troponin I (TnI) degradation and sensitivity to Ca^2+^ [34]. Moreover, this protection of cardiac function is associated with decreased release of TnI (a clinical marker of myocardial tissue damage).

Taken together, our results further support the literature suggesting that, contrary to chronic LF-EMF exposure, acute exposure to LF-EMFs can have beneficial effects and important cardioprotective actions, namely, by conferring mechanical and cellular protection against I/R. Although further studies are required to elucidate the molecular mechanisms behind cardioprotection by LF-EMFs, the data gathered thus far validates its safety and usefulness, rendering it of possible clinical importance.

## Figures and Tables

**Table 1:** Estimated values of magnetic induction in relation to distance from the head of the applicator.

Value of magnetic induction in relation to distance from the head of applicator:	Induction at value “12”	Induction at value “10”
*d*_1_ = 0 mm → *B*_1_ = 100%	13,440 *μ*T	11,200 *μ*T
*d*_2_ = 3 mm → *B*_2_ = 50%	6,720 *μ*T	5,600 *μ*T
*d*_3_ = 6 mm → *B*_3_ = 18%	2,419 *μ*T	2,016 *μ*T
*d*_4_ = 9 mm → *B*_4_ = 3%	403 *μ*T	336 *μ*T

The max values are indicated in the table for control unit levels 10 and 12.

*d*_1–4_: distance to the head of the applicator.

*B*_1–4_: magnetic field.
